# Deep investigation on inorganic fraction of atmospheric PM in Mediterranean area by neutron and photon activation analysis

**DOI:** 10.1186/1752-153X-7-173

**Published:** 2013-11-06

**Authors:** Pasquale Avino, Geraldo Capannesi, Maurizio Manigrasso, Alberto Rosada, Mario Vincenzo Russo

**Affiliations:** 1 DIPIA, INAIL settore Ricerca, Certificazione e Verifica, Via IV Novembre 144, Rome 00187, Italy; 2 UTFISS CATNUC, ENEA, Via Anguillarese 301, Rome 00123, Italy; 3 Department of Environment, Food and Agriculture, University of Molise, Via De Sanctis, Campobasso 86100, Italy

**Keywords:** Element, PM10, PM2.5, Atmosphere, Urban air, Enrichment factor, INAA, IPAA

## Abstract

**Background:**

Anthropogenic activities introduce materials increasing levels of many dangerous substances for the environmental quality and being hazardous to human health. Major attention has been given to those elements able to alter the environment and endanger human health.

The airborne particulate matter pollutant is considered one of the most difficult task in environmental chemistry for its complex composition and implications complicating notably the behavior comprehension. So, for investigating deeply the elemental composition we used two nuclear techniques, Neutron Activation Analysis and Photon Activation Analysis, characterized by high sensitivity, precision and accuracy. An important task has been devoted to the investigation of Quality Control (QC) and Quality Assurance (QA) of the methodology used in this study.

This study was therefore extended as far back as possible in time (from 1965 until 2000) in order to analyze the trend of airborne concentration of pollutant elements in connection with the industrial and lifestyle growth during the entire period.

**Results:**

Almost all the elements may be attributed to long-range transport phenomena from other natural and/or anthropogenic sources: this behavior is common to all the periods studied even if a very light decreasing trend can be evidenced from 1970 to 2002. Finally, in order to investigate a retrospective study of elements in PM10 and their evolution in relationship with the natural or anthropogenic origins, we have investigated the Enrichment Factors. The study shows the EF trends for some elements in PM10 during four decades.

**Conclusions:**

The two nuclear techniques have allowed to reach elevated sensibility/accuracy levels for determining elements at very low concentrations (trace and ultra-trace levels). The element concentrations determined in this study do not basically show a significant level of attention from a toxicological point of view.

## Background

One of the most interesting and difficult task in environmental chemistry is the investigation of the inorganic chemical composition of the particulate matter. Different reasons are the basis of this consideration: for instance, the very low levels of some elements (e.g., Cd, Hg, Se, at ultra-trace levels), the sampling collection which is more representative as possible, the availability of analytical methods (expecially, no-destructive but really sensitive methodologies) [1-11].

The element distribution in air is fundamentally determined by resuspension from soil and water of various substances of natural and/or artificial origin, by their type of circulation due to the meteorological events and by the chemical element behavior. Anthropogenic activities introduce species increasing levels of many substances which may endanger the environmental quality and represent a hazard to human health. Major attention has been given to those elements able to alter the environment and endanger human health.

The issue regarding the determination of trace elements in airborne particulate have a preeminent position due to the presence of some toxic elements (e.g., Cd, Hg and Pb): further, the airborne particulate matter enters into the climate global change problem, giving place to increasing global levels that may affect widely the biological systems.

The very complex composition and implications of the airborne particulate matter pollutant PM_10_ and, expecially, the distribution and multielemental composition of the fine fraction (i.e., particles with diameter <2.5 μm, PM_2.5_), complicate notably the behavior comprehension. The evaluation of background levels due to natural pathways of circulation, seems the preliminar action to be undertaken. This study was therefore extended as far back as possible in time (from the seventies until nowdays) in order to analyze the trend of airborne concentration of pollutant elements in connection with the industrial and lifestyle growth during the entire period.

Instrumental nuclear techniques are widely used in this field [12] as they represent the most reliable method for analyzing trace and/or ultra-trace elements in air particulate PM_10_ and PM_2.5_. Instrumental Neutron Activation Analysis (INAA) as well Instrumental Photon Activation Analysis (IPAA) have been employed in this work to measure interesting toxicologically elements. INAA is not universally applicable with regard to the elements that can be determined, as the technique does not have sufficient sensitivity for Ca, Ti, Sr and Zr for example, and Nb, Pb, Tl and Y are impossible to measure. Similarly, determination of some elements is complicated due to interfering nuclear reactions or from the products of uranium fission (samples with elevated U concentration) [13,14]. An useful complementary method to INAA is Instrumental Photon Activation Analysis (IPAA), which enables the determination of the above elements. The IPAA method has been utilised on a much smaller scale in analytical field [15,16]. In IPAA, the nuclei can be activated through photonuclear process: in fact, contrary to INAA based mainly on the neutron capture reaction (n, γ), PAA employs photonuclear reactions, particularly the (γ, n) reaction [17]. Activation is induced by high energy photons having energy of at least 10 MeV. The photonuclear reaction data of the elements require around 30 MeV for activation energy. For analytical data interpretation, IPAA results have been considered only for the elements that cannot be determined and/or are difficult to determine by INAA [18-27], and for which IPAA provides results of comparable or better quality. Determination of other elements, such as Mn, Rb, Cs, Ba, Ce, U by IPAA should be considered of subsidiary value because INAA is more sensitive. Finally, as reported in literature [28], IPAA is not as commonly used as INAA but possibilities for element determination by both INAA and IPAA depend strongly on the matrix composition.

In particular, over As, Cd, Cr, Hg, Pb, Sb and Zn, i.e. metals considered of greater health concern, other elements, e.g. Au, Cs, La, Mo, Sc, Se, Sm, Th, W, were measured.

## Results and discussion

### Quality control

Table 1 shows the analytical quality control performed on a Standard Reference Material (SRM). This control was performed through an intercomparison campaign for 14 elements promoted by the International Atomic Energy Agency (IAEA) on air filter samples among different worldwide laboratories (130) using both spectrochemical (colorimetry, fluorescence, x-ray fluorescence, infrared spectrometry, atomic absorption and emission spectrometry, ICP-AES, ICP-MS), electrochemical (polarography, voltammetry) and nuclear (INAA, IPAA, isotopic dilution, beta counting) analytical techniques. For each element our values (“measured value”) and the “certified value” are reported: the third column (“average value”) represents the value averaged among all the determinations performed by different laboratories interested in the round-robin. As it can be noted, the agreement between our and the real value is quite good except for some elements such as Ba, U and Zn. For Ba and U this discrepancies can be due to the difficulty to analyze such kind of elements even if for Ba our “measured value” (43.4 ± 0.5) and the “average value” (39.05 ± 15.62) are quite similar. For Zn the situation is little bit different. The “measured value” (132 ± 18 μg g^-1^) falls out the certified value (152 μg g^-1^) whereas it is in “average value” range (141 ± 16 μg g^-1^): the two-tailed P value is less than 0.001 and, by conventional criteria, this difference is considered to be statistically significant.

**Table 1:** **Results of the Quality Control on IAEA air filter samples (μg g**^**-1**^**)**

**Element**	**Measured value**	**Certified value**	**Average value**	
	**mean ± s.d.**	**mean**	**mean**	**2σ (%)**
As	4.9±0.5	5.6	4.59	43
Au	1.26±0.10	1.15	1.06	21
Ba	43.4±0.5	53.8	39.05	40
Cd	10.6±1.0	9.96	9.8	18
Co	1.3±0.1	1.12	1.18	38
Cr	4.7±0.8	5.6	4.8	13
Cu	51.6±0.5	48.8	44.8	16
Fe	193±17	207.9	200.1	8
Mn	31.2±1.0	31.9	30.1	14
Mo	1.26±0.2	1.14	1.56	70
Se	1.01±0.10	1.06	1.01	11
U	0.78±0.10	1.02	0.99	14
V	8.04±0.35	8.00	7.2	16
Zn	132±18	152	141	12

The “measured value” is the average of seven determinations on seven different replicates. s.d.: standard deviation.

### Particulate matter results

Average concentration values, minimum and maximum levels and standard deviation of the elements determined in the PM_2.5_ fraction are shown in Table 2 whereas the correlation coefficients of the analyzed elements are reported in Table 3. It should be noted that many elements cannot be possible to determine in these samples: the main reasons depend on both the granulometric size fraction, 2.5 μm, as just reported in literature [9], and the very low levels reached by some elements (e.g., Nd is below LOD). Basically, the concentration levels of the elements are very low. There is only a very significant correlation between Br and Sb (correlation coefficient 0.95). The Br presence in air is essentially attributed to natural (e.g., marine aerosol) and anthropogenic (e.g., autovehicular traffic [7] sources. This high correlation coefficient with Sb hypothesizes a strong anthropogenic contribution to the Br level. Furthermore, it can be noted a wide scattering between the correlation coefficients: only As, Co, Fe, Sc, Sb, Se are highly correlated (0.7 < correlation coefficient < 0.9, marked in bold in Table 3) whereas Au, Ba, Br, Ce, Cr, Cs, La, Sm result scarcely correlated. This behavior can be expected considering the chemical-physical properties of the elements and the granulometric size (<2.5 μm), as previously evidenced in previous papers [7,9].

**Table 2:** **Synoptic table (mean value, min-max values and standard deviation) of elements concentration (ng m**^**-3**^**) determined in PM**_**2.5 **_in downtown Rome (LOD: limit of detection; * expressed as pg m^**-3**^; ** expressed as μg m^**-3**^**)**

**Element**	**PM2.5**		
	**Mean**	**Min-Max**	**St. dev.**
As	1.06	0.121-2.76	0.044
Au	0.009	0.000-0.050	0.012
Ba	3.76	1.91-6.45	2.38
Br	17.1	3.20-50.4	13.9
Ce	0.130	0.033-0.335	0.089
Co	0.167	0.077-0.331	0.065
Cr	3.03	1.29-6.40	1.30
Cs	0.047	0.004-0.124	0.037
Eu*	1.14	1.12-1.16	0.029
Fe**	0.074	0.005-0.212	0.059
Hf	0.018	0.006-0.032	0.010
Hg	0.818	0.195-2.12	0.655
La*	22.6	8.73-53.3	10.5
Mo	0.748	0.017-3.04	0.699
Nd	<LOD		
Ni	3.54	1.91-5.82	1.45
Rb	1.82	0.416-3.74	1.07
Sb	3.60	0.690-12.6	3.24
Sc*	3.14	0.208-7.49	2.41
Se	0.567	0.116-1.55	0.415
Sm*	3.88	0.208-7.78	2.13
Th	0.027	0.007-0.041	0.010
W	0.636	0.062-2.86	0.682
Yb	0.011	0.003-0.027	0.007
Zn	58.0	4.78-252	61.3

**Table 3:** **Correlation coefficients for the trend of concentrations of analyzed elements present in PM**_**2.5**_

**As**	**Au**	**Ba**	**Br**	**Ce**	**Co**	**Cr**	**Cs**	**Fe**	**Hf**	**Hg**	**La**	**Mo**	**Rb**	**Sb**	**Sc**	**Se**	**Sm**	**Th**	**W**	**Zn**	
	0.25	-0.80	**0.85**	0.37	**0.85**	0.65	0.50	**0.80**	0.51	-0.35	**0.70**	0.50	0.39	**0.83**	0.40	**0.80**	0.55	0.20	-0.19	0.37	**As**
		-0.97	0.58	0.51	0.35	0.06	0.54	0.33	**0.73**	0.35	0.01	0.10	**0.77**	0.48	0.01	0.56	0.08	-0.32	-0.19	0.16	**Au**
			-0.97	-1.00	-0.98	-0.95	-0.89	-0.65	-1.00	-0.43	-0.81	-0.91	-1.00	-0.99	0.62	-0.94	-0.98	-0.98	-0.11	-0.65	**Ba**
				0.61	**0.87**	0.54	0.57	**0.83**	**0.86**	-0.22	0.57	0.23	0.68	**0.95**	0.35	**0.89**	0.50	0.16	-0.17	0.58	**Br**
					0.51	0.39	0.29	0.46	0.59	0.15	0.41	0.15	**0.77**	0.64	0.05	0.66	0.10	-0.32	-0.14	0.50	**Ce**
						0.69	0.53	**0.81**	0.63	-0.22	**0.72**	0.24	**0.78**	**0.90**	0.35	**0.86**	0.53	0.25	0.10	0.60	**Co**
							0.09	0.67	-0.13	-0.34	0.66	0.43	0.45	0.61	0.29	**0.71**	0.45	0.04	-0.24	0.44	**Cr**
								0.44	**0.83**	0.15	0.47	0.19	**0.72**	0.60	0.13	0.43	0.19	0.18	-0.20	0.05	**Cs**
									0.39	-0.38	**0.73**	0.14	0.57	**0.85**	0.65	**0.79**	**0.72**	0.27	-0.29	0.50	**Fe**
										0.56	0.13	0.48	-0.05	**0.82**	0.26	**0.73**	0.46	0.26	0.01	0.55	**Hf**
											-0.34	-0.16	0.22	-0.17	-0.41	-0.21	-0.40	-0.37	-0.03	-0.04	**Hg**
												0.30	0.49	0.64	0.42	0.60	0.55	0.41	-0.15	0.26	**La**
													0.36	0.27	-0.07	0.40	0.12	-0.31	-0.21	0.03	**Mo**
														**0.79**	-0.34	**0.76**	-0.29	-0.57	-0.02	0.37	**Rb**
															0.41	**0.87**	0.53	0.15	-0.13	0.68	**Sb**
																0.24	**0.89**	0.47	-0.11	0.44	**Sc**
																	0.44	-0.06	-0.28	0.53	**Se**
																		0.39	-0.09	0.50	**Sm**
																			0.24	0.17	**Th**
																				0.07	**W**
																					**Zn**

In Tables 4 and 5 are reported for each element both the summer and winter average levels as well as the maximum and minimum values for each season measured in PM_10_ samples by INAA and IPAA. The samples were collected in outskirt of Rome and downtown [29], respectively, during summer and winter period. It may be noted that elements of anthropogenic origin show winter concentration levels higher than the summer ones, probably owing to an enhanced production in the winter period; in contrast elements of natural origin show summer concentration levels higher than the winter ones, possibly as a consequence of an increased resuspension of soil matter in summer. The values in the two tables are quite similar for some elements whereas difference distributions between the two time periods are evident. This last issue influences the element behavior in atmosphere.

**Table 4:** **Seasonal element concentrations (average, min and max levels expressed as ng m**^**-3**^**; a: pg m**^**-3**^**) in PM**_**10 **_**determined by INAA and IPAA in atmospheric particulate sampled in outskirt of Rome**

**Element**	**Summer**			**Winter**		
	**Average**	**Min**	**Max**	**Average**	**Min**	**Max**
Ag	0.42	0.2	0.9	0.31	0.1	0.5
Al	3800	200	14000	1800	400	8500
As	2	0.5	9	3	0.2	15
Ba	4	2	8	13	3	57
Br	14	3	51	5	0.4	18
Ca	2400	300	5800	1800	300	2500
Cd	0.9	0.2	4	0.9	0.2	5
Ce	1	0.09	3	0.7	0.1	4
Cl	1300	300	9300	1400	200	5400
Co	0.4	0.05	2.1	0.3	0.04	2.4
Cr	6	1	12	9	1	21
Cs	0.2	0.01	1.9	0.3	0.01	1.6
Eu^a^	0.15	0.02	0.22	0.11	0.02	0.24
Fe	1850	600	4800	1500	350	4100
Hf^a^	91	21	320	75	11	250
Hg	0.64	0.1	1.6	0.45	0.1	1.5
I	6	2	12	4	3	9
K	930	100	3400	490	50	2800
La^a^	12	1	38	7	0.9	25
Mg	1490	100	3500	950	100	1500
Mn	52	12	98	37	10	85
Mo	3.4	0.4	12	2.8	0.2	11
Na	2500	300	9800	1530	200	5200
Ni	5.5	1	19	4	1	15
Pb	42	12	157	54	15	250
Rb	5	1	18	6	1	24
Sb	8	0.5	17	9	0.4	31
Sc^a^	45	14	120	51	12	135
Se	0.7	0.1	1.3	0.6	0.1	1.6
Ta	0.02	0.01	0.09	0.03	0.01	1.1
Tb	0.048	0.009	0.32	0.035	0.005	0.42
Th^a^	200	22	510	180	19	460
Ti	250	50	930	124	100	630
Tl	1.2	0.3	6	1.7	0.5	9
U	0.3	0.02	0.9	0.2	0.03	0.8
V	15	1	34	21	5	49
Zn	130	25	290	180	40	640
Zr	45	5	75	39	7	61

**Table 5:** **Seasonal element concentrations (average, min and max levels expressed as ng m**^**-3**^**) in PM**_**10 **_**determined by INAA and IPAA in atmospheric particulate sampled in downtown Rome**

**Element**	**Summer**			**Winter**		
	**Average**	**Min**	**Max**	**Average**	**Min**	**Max**
Ag	0.22	0.1	0.5	0.25	0.1	0.6
Al	2900	500	5300	800	200	1600
As	6	1	15	4	1	9
Ba	60	30	120	30	5	70
Br	40	20	70	70	10	140
Ca	2200	800	4200	1200	300	2000
Cd	0.4	0.3	0.9	0.75	0.3	2
Ce	5.6	0.7	10	2	0.2	5
Cl	1300	300	3900	1400	300	4700
Co	0.7	0.4	1.2	0.5	0.1	0.9
Cr	7	4	13	16	2	38
Cs	1.2	0.6	2.2	0.6	0.2	1.3
Eu	0.08	0.04	0.14	0.03	0.01	0.07
Fe	2200	1000	3600	1100	400	2700
Hf	0.36	0.15	0.62	0.13	0.01	0.30
Hg	0.14	0.05	0.36	0.13	0.03	0.20
I	6	3	8	6	3	10
K	1120	50	1900	320	50	1000
La	5	2	9	2	0.3	4
Mg	1090	50	2700	560	200	1200
Mn	40	15	60	35	10	90
Mo	0.5	0.2	0.6	2	0.3	7
Na	1500	300	7000	730	200	3500
Ni	1.7	1	5	8	3	13
Pb	120	80	200	270	80	840
Rb	17	10	30	7	2	15
Sb	2	0.7	4	2	0.7	7
Sc	0.3	0.1	0.5	0.1	0.03	0.2
Se	0.7	0.2	1.1	0.5	0.2	1.3
Ta	0.03	0.01	0.05	0.01	0.005	0.02
Tb	0.055	0.1	0.10	0.024	0.01	0.05
Th	3	1	5	0.9	0.3	2.5
Ti	540	200	1050	265	200	500
Tl	1	0.5	2	1.4	0.5	4
U	0.4	0.1	0.7	0.2	0.06	0.4
V	8	1	15	14	8	23
Zn	110	50	260	190	50	520
Zr	50	10	90	20	5	50

As a very simple approach for understanding the element behavior, they were grouped into three categories according to the value of the ratio (R) of summer to winter average levels (Table 6). The first group includes elements whose R is greater than 2; the second group elements whose R is less than 2 but greater than 0.5; the last group includes elements whose R is less than 0.5. Looking at the table, it can be noted a ratio difference between the elements determined in outskirt and in downtown: only 6 elements, such as Al, Br, K, Na, Ti, U, show R > 2 in samples collected in downtown whereas 20 elements, i.e. Al, Ba, Ca, Ce, Cs, Eu, Fe, Hf, K, La, Mg, Na, Rb, Sc, Ta, Tb, Th, Ti, U, Zr, show ratios > 2 in samples collected in outskirt.

**Table 6:** **Grouping of elements in PM**_**10 **_**according to the ratio of summer to winter seasonal average**

**Ratio >2**	**Ratio ~1**	**Ratio <1**
Outskirt		
Al, Ba, Ca, Ce, Cs, Eu, Fe, Hf, K, La, Mg, Na, Rb, Sc, Ta, Tb, Th, Ti, U, Zr	Ag, As, Br, Cl, Co, Hg, I, Mn, Sb, Se, Tl	Cd, Cr, Mo, Ni, Pb, V, Zn
Downtown		
Al, Br, K, Na, Ti, U	Ag, Ca, Cd, Ce, Co, Eu, Fe, Hf, La, Mg, Mn, Mo, Ni, Se, Tb, Th, Zr	As, Ba, Cl, Cr, Cs, I, Pb, Rb, Sb, Sc, Ta, Tl, V, Zn

Elements of natural origin are only found in the first group, while elements of both natural and anthropogenic origin are present in the second group. The third group includes only pollutant elements (Cd, Cr, Mo, Ni, Pb, V, Zn).

An intersting considerations, coming from the data analysis, are obviously strictly related to the general meteorological characteristics of Italy and therefore contain some peculiarity. If they are compared to similar results obtained in other countries under different meteorological conditions, it can be seen that they agree fairly well for the pollutant elements, whereas for most elements of natural origin there are sensible differences that may be related to the geomorphological and meteorological characteristics. In fact, the higher values found in this study for Al, Cs, Na, Rb, Th, Ti, U and rare earths are to be related to the element content of the volcanic rocks which are very widespread in Latium [30,31].

### The enrichment factor (EF) application

**Figure 1:**
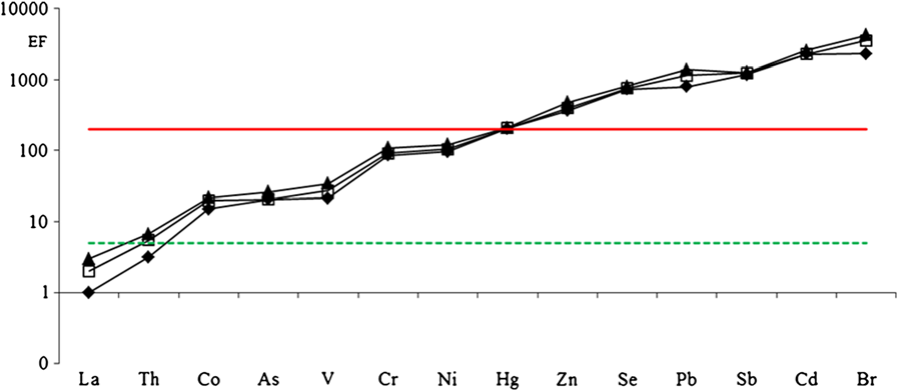
**EF trend comparison of selected trace elements in PM10 fraction calculated using La as normalizing element.** ▲: period 1965–78; □: period 1989–92; ♦: period 2000–05.

In order to investigate a retrospective study of elements in PM_10_ and their evolution in relationship with the natural or anthropogenic origins, Table 7 reports the levels of selected elements collected in the last 4 decades: the data obtained show a decrease ranging between 24% (Co) and 91% (La), except for Hg, Sb and Se. Basically, this may be attributed to the technological growth during the entire period and to the adoption of anti-pollution system in domestic heating and in industrial plants. Mercury decreases slowly (during the four decades the values are almost similar ranging between 0.09 and 0.13) whereas Se increases except during the period 1989–92 (probably due to the sample site choice), and Sb is 4-times higher than the seventies. For a better knowledge of this evolution and, expecially, of the element origin, we have calculated the enrichment factors (EFs) with respect to the element abundance in the upper continental crust. Elements with EF values much higher than 1 can be considered of non-crustal origin and may be attributed to long-transport phenomena from other natural and/or anthropogenic sources. The EFs have been calculated in according to the equation reported in refs. [7,32] and La as normalizing element [21]. Figure 1 shows the EF trend for selected elements in PM_10_ during four decedes. As can be noted, all the elements may be attributed to long-range transport phenomena from other natural and/or anthropogenic sources: this behavior is common to all the period studied even if a very light decreasing trend can be evidenced from 1970 to 2005.

**Table 7:** **Levels (ng m**^**-3**^**) of selected elements in PM**_**10 **_**investigated along four decades in downtown Rome**

		**PM**_**10**_	
	**1965-78**	**1989-92**	**2000-05**
As	4.00		1.35
Br	70	50	22
Cd	0.751		0.520
Co	0.498	0.523	0.379
Cr	16	2.3	7.28
Hg	0.131	0.092	1.07
La	2.04	0.803	0.188
Ni	8.01	1.72	4.54
Pb	270	172	92
Sb	1.99	2.13	9.22
Se	0.533	0.091	0.692
Th	0.911	0.723	0.229
V	14	4.82	4.02
Zn	190	28	80

In Figure 1, showing the EFs of some elements, three groups can be identified: La and Th ranging between 1 and 5; Co, As, V, Cr and Ni between 1 and 100; Hg, Zn, Se, Pb, Sb, Cd and Br ranging between 200 and 2500.

Basically, some particular considerations can be extrapolated: the high EF values found for Br (and Pb as well) by both the elaborations could be attributed to the use of leaded gasoline gasoline (cars with leaded gasoline are still present at the end of nineties’); the sources of As, Pb, Sb and Zn would be looked for among the various anthropogenic activities in the Rome area and particularly Sb and Zn could be of traffic origin being essential components of anti-friction alloys and car tires.

**Figure 2:**
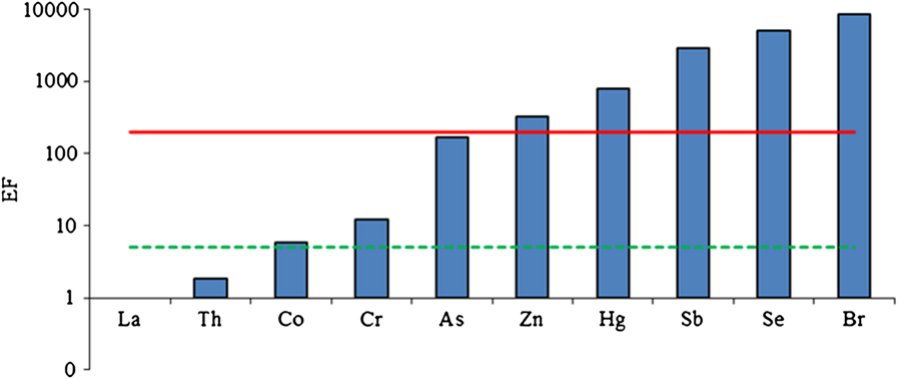
**EFs of selected elements in PM**_**2.5 **_**fraction calculated using La as normalizing element.**

Finally, a same approach has been performed to elements investigated in the PM_2.5_ fraction, even if no historical data series are available. Figure 2 reports the results obtained on the PM_2.5_ fraction: Co, Cr, As, Zn, Hg, Sb, Se and Br show EF values ranging between 5 and 8500, respectively.

It should be noted that the EFs in PM_2.5_ fraction is more elevated that in PM_10_ fraction, expecially for Br, Se, Sb and Hg: this could be due to the different granulometric size and the different ability to penetrate of such elements. This occurrence can be an index of the different bioavailability of an element series present in PM_2.5_ fraction compared to PM_10_ fraction. As reported above, the higher EF value found for Br and attributed to the use of leaded, is more evident in this fraction.

## Conclusions

The experimentation has been addressed for getting the maximum analytical informative ability from the single sample determinations. The INAA and IPAA techniques allow to reach such elevated sensibility/accuracy levels to furnish discreet values for elements present at very low concentrations (trace and ultra-trace levels). In particular, IPAA is a unusual technique but its coupling with INAA, another nuclear methodology, can be useful for solving a lot of analytical problems.

The element concentrations determined in this study do not seem to show significant level of attention from a toxicological point of view, stressing preliminary results obtained in previous studies [7,9]. On the other hand, the element behavior trend during these last 40 years in downtown Rome is lightly decreasing although their main source is the anthropogenic activities.

## Experimental

### Sampling

The sample collection has been performed in a large period of time, almost 40 years: this means that the operation sampling has been carried out by means of different samplers. During the seventies the sampling occurred with no cut-off whereas since nineties the sampling heads have been introduced allowing to separate different granulometric fraction (firstly PM_10_ and after PM_2.5_). In particular, the latter campaigns were performed by a dichotomous sampler (mod. SA 241, Graseby,-Andersen) operating at 16.7 L min^-1^. This sampler has a PM10 size selective inlet and separates the aerosol into fine (aerodynamic diameter, D_a_, <2.5 μm) and coarse (2.5 μm < D_a_ < 10 μm) fractions. Particulate matter was collected on polymethylpentane ringed, 2.0 μm pore size, 37 μm, Teflon membranes (Gelman, type R2PJ). This sampler has been designed as reference by USA-EPA. The PM_2.5_ samples were stored in box at controlled conditions (atmosphere and temperature).

About 300 air samples have been collected in downtown Rome (via Urbana, an area characterized by high presence of anthropogenic activities) and Roman outskirt (Anguillara area, a rural site); all the sampling were performed at ground level. The sampling was 24-hr long for each filter. All the storage and handling sample treatment were carried out at the ENEA and ISPESL laboratories.

### INAA and IPAA analyses

Among the different analytical methodologies available for element determination we used nuclear approach for its important analytical properties [7,9,20,21,33]. In fact, the various analytical techniques (spectroscopy, electrochemistry, chromatography, bioanalytical methods) [34,35] do not permit to have the maximum information because of their limitations. INAA is well known as reference analytical technique because all the experimental steps are totally traceable and there is absence of physical-chemical sample manipulation reducing the positive and/or negative artifacts formation [7,9,20,21]. Furthermore, because of its high sensitivity, multi-elemental character allowing the determination of about 40 elements with a good Limit of Detection (LOD) [36] (Table 8) and accuracy, the INAA has surpassed other instrumental methods for trace/ultra-trace metal and rare hearths analysis: a comparative study [37-41], however, has pointed out that INAA is blank free and expecially suitable for the analysis of reference materials [41]. Further, we used IPAA as a complementary technique for determining elements: in particular, Pb, Tl and Zr which are difficult to determine by INAA, are important from toxicological and environmental point of view.

**Table 8:** Nuclear data and LOD of elements by INAA and IPAA (m: minutes; h: hours; d: days; y: years)

**Element**	**Product nuclide**	**Half life**		**γ-Ray used (keV)**	**LOD (g)**
*INAA*					
Ag	^110m^Ag	253.0	d	657.8	1×10^-10^
Al	^28^Al	2.3	m	1778.9	3×10^-8^
As	^76^As	26.3	h	559.2	1×10^-12^
Au	^198^Au	2.70	d	411.8	6×10^-14^
Ba	^131^Ba	11.5	d	496.3	2×10^-10^
Br	^82^Br	1.47	d	776.5	2×10^-12^
Ca	^49^Ca	8.8	m	3083.0	2×10^-7^
Cd	^115m^In	53.0	h	336.6	3×10^-11^
Ce	^141^Ce	32.38	d	145.4	8×10^-10^
Cl	^38^Cl	37.3	.	1642.0	2×10^-9^
Co	^60^Co	5.272	y	1332.5	6×10^-11^
Cr	^51^Cr	27.7	d	320.0	1×10^-10^
Cs	^134^Cs	2.062	y	795.7	1×10^-11^
Eu	^152^Eu	12.7	y	1408.0	2×10^-12^
Fe	^59^Fe	45.1	d	1099.2	2×10^-8^
Hf	^181^Hf	42.4	d	482.2	2×10^-11^
Hg	^203^Hg	46.9	d	279.0	4×10^-11^
I	^128^I	25.0	m	442.7	8×10^-11^
K	^42^K	12.52	h	1524.7	2×10^-10^
La	^140^La	40.27	h	1596.2	1×10^-12^
Mg	^27^Mg	9.5	m	1014.1	2×10^-8^
Mn	^56^Mn	2.58	h	1810.7	8×10^-13^
Mo	^99^Mo	2.75	d	141.0	3×10^-11^
Na	^24^Na	15.0	h	1368.4	4×10^-12^
Nd	^147^Nd	11.1	d	531.0	1×10^-11^
Ni	^58^Co	70.78	d	810.7	6×10^-9^
Rb	^86^Rb	18.66	d	1076.7	8×10^-10^
Sb	^122^Sb	2.70	d	564.0	1×10^-12^
Sc	^46^Sc	83.85	d	889.2	2×10^-12^
Se	^75^Se	120.4	d	264.6	1×10^-10^
Sm	^153^Sm	1.948	d	103.1	6×10^-13^
Ta	^182^Ta	115	d	1221.6	1×10^-11^
Tb	^160^Tb	72.1	d	879.4	2×10^-12^
Th	^233^Pa	27.4	d	311.8	1×10^-11^
Ti	^51^Ti	5.8	m	320.0	1×10^-8^
U	^239^Np	2.35	d	228.2	5×10^-12^
V	^52^V	3.76	m	1434.4	9×10^-10^
W	^187^W	24.0	h	685.7	1×10^-12^
Yb	^175^Yb	4.21	d	396.1	1×10^-12^
Zn	^65^Zn	243.8	d	1115.5	1×10^-10^
*IPAA*					
As	^76^As	1.097	d	559.1	10^-7^
Ca	^47^Ca	4.536	d	1297.1	10^-7^
Ce	^141^Ce	32.5	d	145.4	10^-7^
Cr	^51^Cr	27.7	d	320.0	10^-8^
Cs	^134^Cs	2.062	y	795.9	10^-8^
Mn	^54^Mn	312.2	d	834.8	10^-6^
Nb	^92m^Nb	10.15	d	934.5	10^-8^
Pb	^204^Pb	52.1	h	279.0	10^-7^
Sr	^85^Sr	64.84	d	514.0	10^-6^
Ti	^47^Sc	3.341	d	159.4	10^-8^
Tl	^203^Tl	12.0	d	440.0	10^-7^
Y	^88^Y	106.61	d	1836.1	10^-7^
Zr	^90^Zr	79.4	h	909.0	10^-8^

LODs calculated according to ref. [36] for matrix free elements, i.e. without disturbing activities from a sample matrix.

Table 8 shows the nuclear data (as product nuclide, half-life and energy peak emission) and LOD (expressed as ng m^-3^) of each element investigated in this study by means of INAA and IPAA. It should be noted the very low LODs reached by nuclear techniques in relation with other analytical methodologies [20,22].

**Figure 3:**
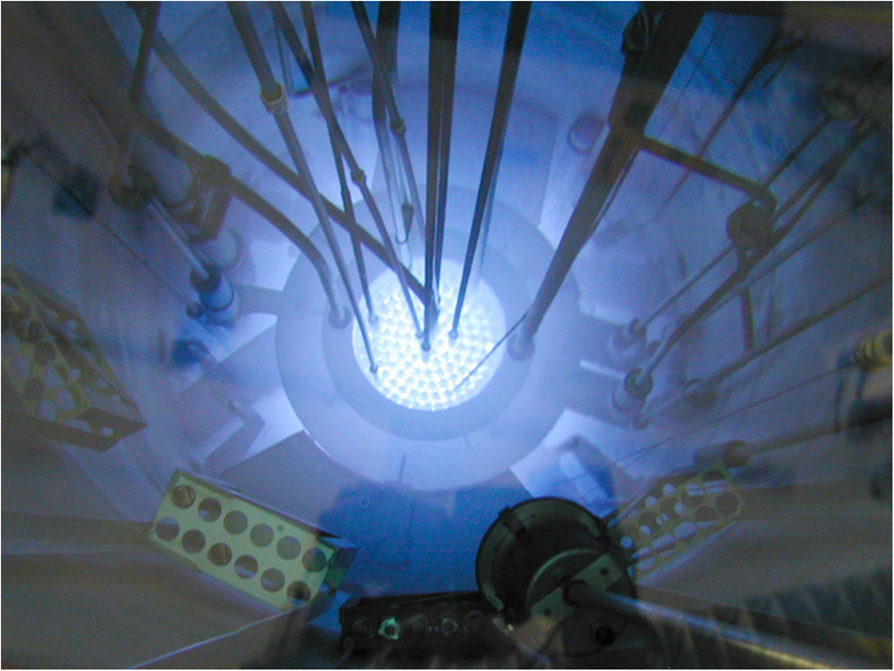
Triga nuclear reactor (1 MW) core with Cherenkov effect.

INAA - Samples, blank and standards, put in nuclear-grade polyethene cylinders (Kartell), were irradiated at a neutron flux of 2.6×10^12^ n×cm^-2^×s^-1^ for 32.55 h in rotatory rack “Lazy Susan” of the nuclear reactor Triga Mark II of the ENEA-Casaccia Laboratories (Figure 3) [42].

For the analysis primary and secondary standards were used. Primary standards (Carlo Erba, Milano, Italy) were As, Cd, Co, Cr, Cs, Fe, Hg, La, Ni, Sb, Se, Sm and Zn whereas as secondary standards United States Geochemical Survey (U.S.G.S.) nn. 1, 4, 6 and the Coal Fly Ash (NIST) n. 1633a were used.

**Figure 4:**
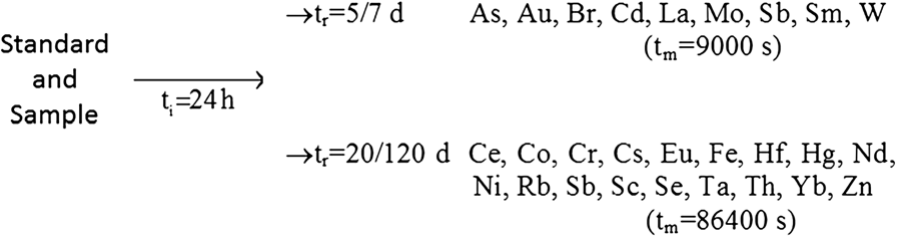
**Scheme for INAA analysis of standards and samples (t**_**i**_**: irradiation time; t**_**r**_**: cooling time; t**_**m**_**: measurement time).**

After irradiation, γ-ray spectrometry measurements of different duration (Figure 4) were carried out using a HPGe detector (FWHM 1.68 keV at 1332 keV) connected to a multichannel analyzer equipped with software packages for a γ-spectra analysis.

A first measurement series was performed 5/7 days after the end of irradiation with measurement times of 3000 and 9000 s for sample for determining As, Au, Br, Cd, La, Mo, Sb, Sm, W [7,13,40]. The second series was performed 20/120 days after the end of irradiation with measurement times of 24-100 h for sample for determining Ce, Co, Cr, Cs, Eu, Fe, Hf, Hg, Nd, Ni, Rb, Sb, Sc, Se, Ta, Th, Yb, Zn [7,25,40].

IPAA - Samples, blank and standards (NIST SRM 1571) were irradiated in the photon beam of the INFN Frascati National Laboratory Linear Accelerator (LINAC) at an average beam current of 40 μA, maximum electron energy of 300 MeV and a W converter of 0.3 mm thickness.

Two series of measurements were carried out: after 36/70 hours As, Ca, Pb, Ti and Zr were measured for 2 hours whereas after 20 days from irradiation Ce, Cr, Cs, Mn, Nb, Sr, Tl and Y were counted for 4 hours.

## Competing interests

The authors declare that they have no competing interests.

## Authors’ contributions

PA, MVR and GC coordinated the study. PA and AR set up the analytical procedure using INAA. MM processed data and provided the comparison with other literature. PA and AR edited the text and prepared the final draft of the paper. GC and MVR approved the final version. All the authors have read and approved the final manuscript.
